# Alternative types of molecule-decorated atomic chains in Au–CO–Au single-molecule junctions

**DOI:** 10.3762/bjnano.6.141

**Published:** 2015-06-19

**Authors:** Zoltán Balogh, Péter Makk, András Halbritter

**Affiliations:** 1 Department of Physics, Budapest University of Technology and Economics and MTA-BME Condensed Matter Research Group, Budafoki ut 8, 1111 Budapest, Hungary

**Keywords:** atomic chains, break junction, carbon monoxide, correlation analysis, gold

## Abstract

We investigate the formation and evolution of Au–CO single-molecule break junctions. The conductance histogram exhibits two distinct molecular configurations, which are further investigated by a combined statistical analysis. According to conditional histogram and correlation analysis these molecular configurations show strong anticorrelations with each other and with pure Au monoatomic junctions and atomic chains. We identify molecular precursor configurations with somewhat higher conductance, which are formed prior to single-molecule junctions. According to detailed length analysis two distinct types of molecule-affected chain-formation processes are observed, and we compare these results to former theoretical calculations considering bridge- and atop-type molecular configurations where the latter has reduced conductance due to destructive Fano interference.

## Introduction

The break junction method is widely used to establish single-molecule nanowires [1–2]. During its controlled rupture a metallic wire thins down to atomic dimensions and finally breaks forming a nanometer-sized gap between the electrodes. This gap can be bridged by single molecules in a self-organized way. As the microscopic details of such molecular junctions can vary from experiment to experiment, a statistical analysis is necessary. The break junction method allows for the statistical investigation of molecular junctions: by closing the junction the metallic electrodes can be reconnected, and afterwards by stretching and breaking the junction, new molecular junctions can be formed.

During the rupture conductance traces are recorded, i.e., the conductance of the breaking wire is measured as a function of electrode displacement. By repeating the break-junction measurement several thousand times a statistical ensemble of conductance traces are collected, from which a conductance histogram can be plotted. Peaks in the histogram reflect the conductance of typical junction configurations, such as single-atom contacts. After the addition of molecules the formation of single-molecule nanowires is signalled by the appearance of new peaks in the histograms [1–2]. However, to determine the details of the formation of molecular junctions further analysis is required [1–28]. Important information can be obtained by further techniques such as noise [3–4], thermopower [5–6] or inelastic spectroscopy [11–14] measurements. However, it has been recently realized that just by the advanced statistical analysis of the measured conductance traces essential information can be obtained. To this end several data analysis methods have been introduced, such as plateau-length histograms [17–20], two-dimensional conductance–displacement histograms [19,21–23] and correlation histograms [25–28].

In this paper we investigate the formation of Au–CO–Au molecular junctions and show that in the presence of CO two new molecular configurations are formed. To identify them we carry out a statistical analysis going far beyond the simple conductance histogram approach. By combining conditional conductance histograms, two-dimensional correlation histograms (2DCH) [25–27] and two dimensional conductance–displacement histograms (2DCDH) [19,21–22] we are able to narrow down the possible configurations formed during the process of breaking. The comparison of our results with calculations of [29–31] implies that the CO molecule can be either incorporated to gold atomic chains (bridge geometry), or can bind next to the chain (atop geometry). In the latter case the conductance of the atomic chain is substantially lowered as a result of destructive interference effect [29].

## Results

### Conductance histograms and correlation analysis

**Figure 1:**
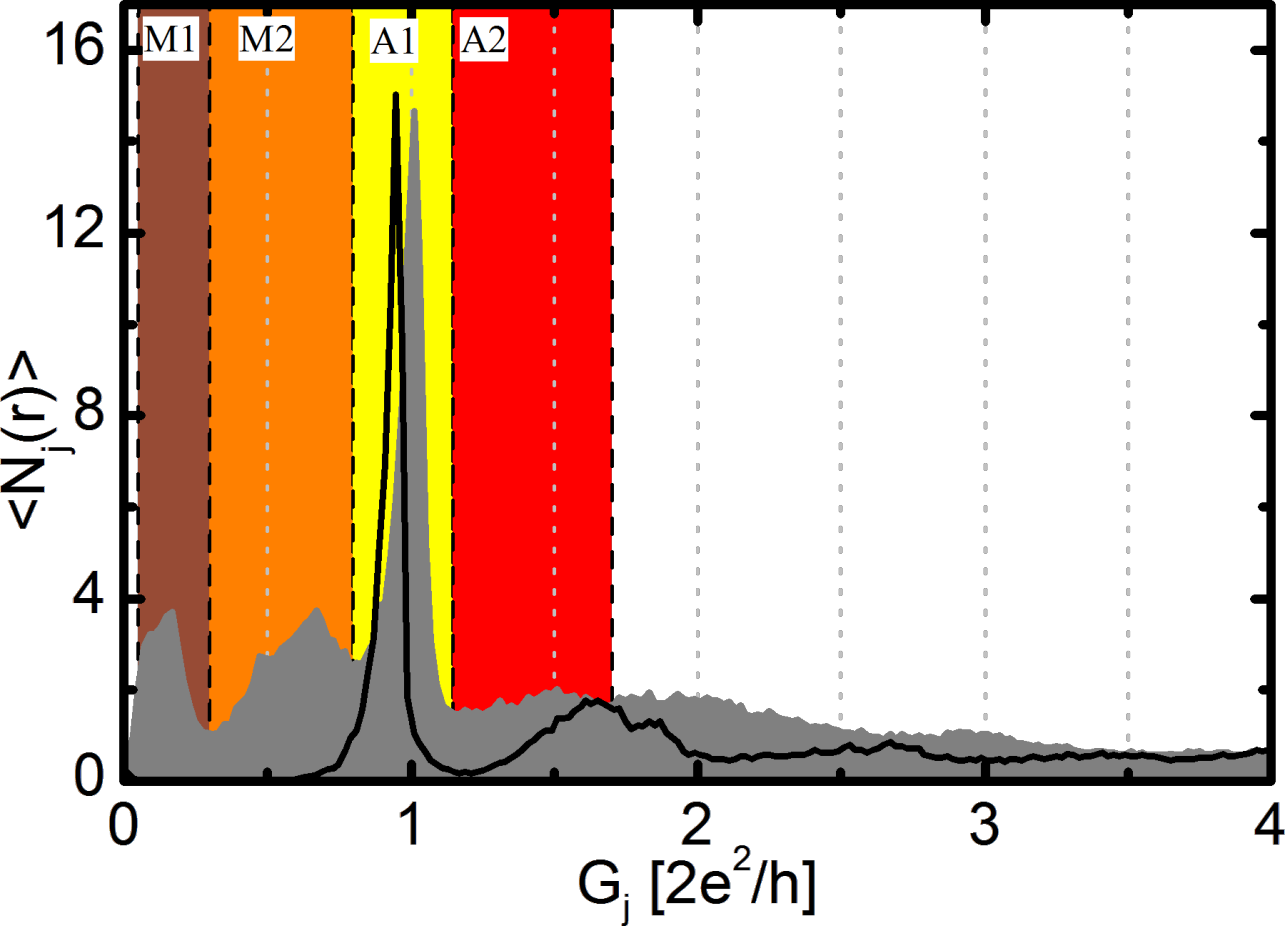
Conductance histogram of Au junctions before (black) and after (grey) the dosing of CO molecules. The latter shows two new molecular peaks at 0.17*G*_0_ and 0.67*G*_0_. The color background shows the important conductance regions discussed in the text: M1 (0.05–0.3*G*_0_, brown), M2 (0.3–0.8*G*_0_, orange), A1 (0.8–1.1*G*_0_, yellow) and A2 (1.1–1.7*G*_0_, red). All histograms are normalized to the number of traces.

The conductance histogram of clean gold junctions at *T* = 4.2 K is shown in Figure 1 with black curve. A sharp peak appears at 1*G*_0_, with smaller peaks at higher conductance, typical for low-temperature measurements on Au junctions [17,32–33]. It is known for gold that after stretching a monoatomic contact it does not necessarily break, but additional atoms can be pulled into the junction forming a nanowire with a single atom cross section and several atoms length [17–18,34–36]. The first peak in the histogram at 1*G*_0_ corresponds both to monoatomic junctions and to atomic chains.

After characterizing clean Au junctions CO molecules are dosed to the junction through a heated metallic tube (see details in Experimental). The conductance histogram of Au–CO junctions is shown in Figure 1 with a grey area graph. The shape of the clean histogram has changed significantly after the dosing of CO: Two new peaks appear in the region below 1*G*_0_ at 0.17*G*_0_ and 0.67*G*_0_. This histogram shows similarities to former measurements on Au–CO junctions [37–38], though in [37] only the molecular peak with higher conductance (approx. 0.7*G*_0_), and in [38] only the one with lower conductance at 0.2–0.3*G*_0_ are reported, whereas in our measurement these two peaks coexist.

To simplify further analysis we define separate conductance regions marked by different colors in Figure 1. The M1 (0.05–0.3*G*_0_, brown) and M2 (0.3–0.8*G*_0_, orange) regions correspond to the two molecular configurations. The region of monoatomic Au junctions and chains is marked by A1 (0.8–1.1*G*_0_, yellow), whereas configurations with a little higher conductance (1.1–1.7*G*_0_, red) are labeled by A2. The latter region is attributed to precursor molecular configurations, as discussed later and in [27]. To gain further information about the nature of these configurations, and their relation to each other we use two dimensional correlation analysis, conditional histograms, and conditional two dimensional conductance–displacement histograms, which are constructed by the proper statistical analysis of the same conductance traces, that are used to plot the conductance histogram itself [19,25–28].

The two dimensional correlation histogram (2DCH) investigates the correlations between different conductance regions. Configurations that tend to appear together during the breaking process induce positive correlations in the 2DCH, whereas configurations excluding each other along a contact rupture induce negative correlations. It can also happen, that both configurations appear, but the lengths of the plateaus within the studied conductance bins exhibit distinct correlations. More details on the technique can be found in [25–26].

**Figure 2:**
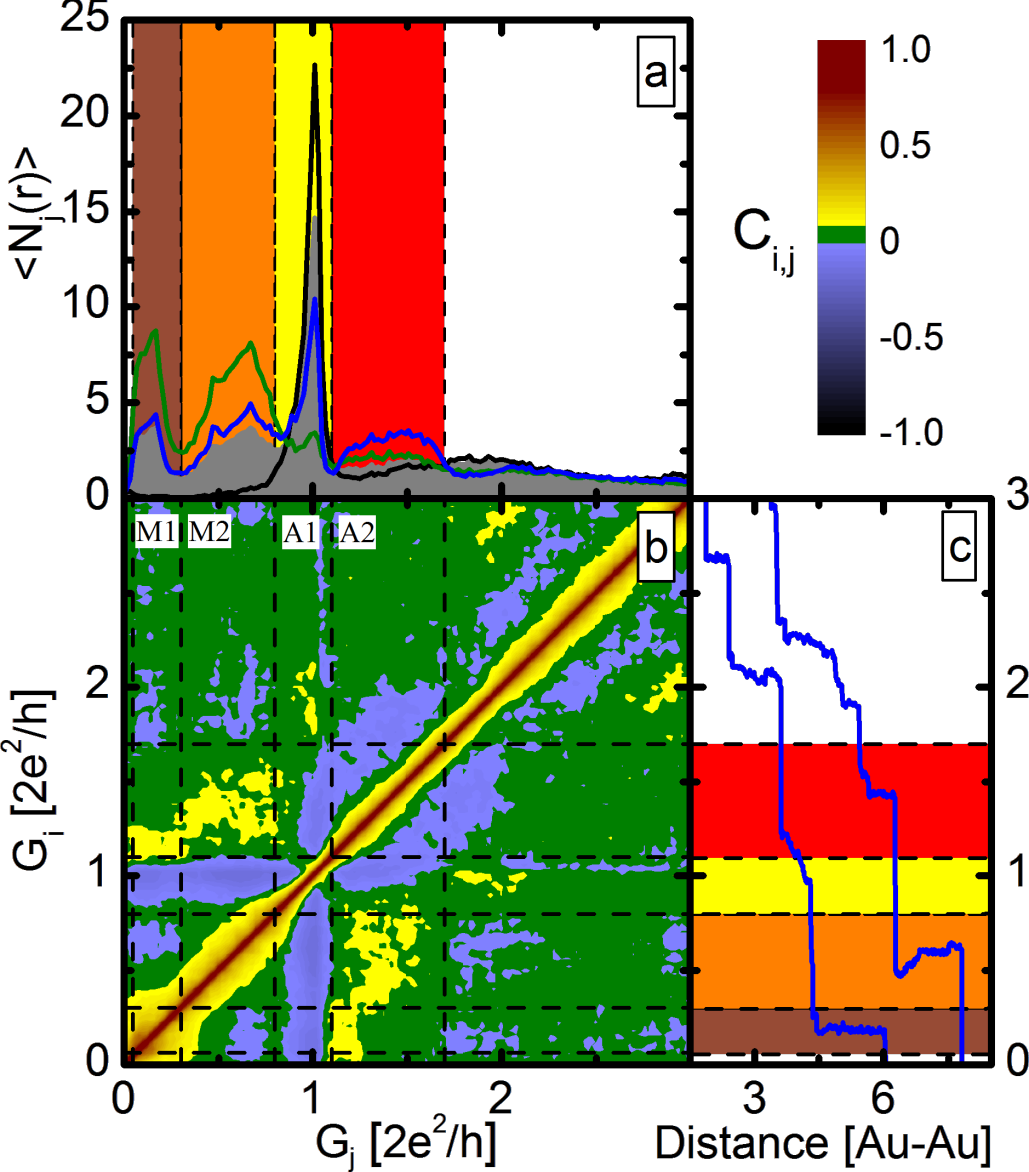
(a) Conditional histograms for selected traces with larger than average length in different regions: M1–M2 (green), A1 (black) and A2 (blue). As a reference the grey area graph shows the histogram for all traces. (b) The 2D correlational histogram of Au–CO junctions. (c) Two demonstrative opening traces.

The 2DCH for Au–CO–Au junctions is shown in Figure 2b. Here the two axes correspond to the two conductances, and the value of the correlation function is shown by colors. Warm colors (yellow-red) correspond to positive correlations, cold colors (blue-black) to negative and green marks to configurations that are independent within the resolution of the method.

The 2DCH in Figure 2b shows clear anti-correlation between the both molecular configurations (M1–M2) and the single atomic configuration (A1). However, the region above 1*G*_0_ (A2), shows positive correlation with both molecular configurations M1–M2. Between the two molecular configurations M1 and M2 an anticorrelation is observed.

Whereas 2DCHs are useful to make a two dimensional map of the relevant correlation effects, to gain a more quantitative picture about the nature and strength of correlations between different regions it is also useful to investigate conditional histograms. Conditional conductance histograms are conductance histograms for breaking traces selected according to a predefined condition. Here, we focus on conductance traces, which are long enough in a predefined conductance region. To construct these histograms, we have determined the average length of breaking traces in the predefined region and selected the conductance traces longer than average. Conditional histograms are normalized to the number of traces included, therefore direct comparison of them is possible. We note here, that the selected data sets obtained by this technique are not distinct, because the length of a given trace can be longer than the average in more regions (positive correlation). More details on the method can be found in [19,26–27].

Conditional conductance histograms selected for different conductance regions are shown in Figure 2a. As a reference, the grey area curve shows the histogram for all traces (5000 curves). The conditional conductance histogram constructed for the A1 region (black) shows the full suppression of the peaks in the molecular region M1–M2. These selected traces correspond to the case, when pronounced (long) plateaus at about 1*G*_0_ are observed. According to the conditional histogram these traces do not show molecular configurations, i.e., the corresponding negative correlation in the 2DCH between A1 and M1–M2, is a strong feature: The A1 and M1–M2 configurations practically exclude each other. A similar feature is seen from the other side as well: The conditional histogram selected for the molecular region M1–M2 (green line) exhibits the almost complete suppression of the peak at 1*G*_0_. This conditional histogram shows an additional interesting feature, namely the enhanced weight in the A2 region compared to the total histogram, which was also reflected by the positive correlation between M1–M2 and A2 in the 2DCH (and similarly the blue conditional histogram for the A2 region exhibits enhanced molecular peaks). All these imply that Au–CO–Au single-molecule junctions are usually not preceded by clean Au monoatomic contacts or atomic chains. There is rather a so-called precursor configuration with higher conductance (A2 region), similarly to our previous report on Ag–CO junctions [27]. We interpret these precursor configurations by the binding of a CO molecule to the side of a single-atom contact, which opens additional conductance channel(s) compared to the single-channel transport in pure Au monoatomic contacts and atomic chains. Figure 2c shows two example traces with a molecular plateau preceded by a precursor configuration.

**Figure 3:**
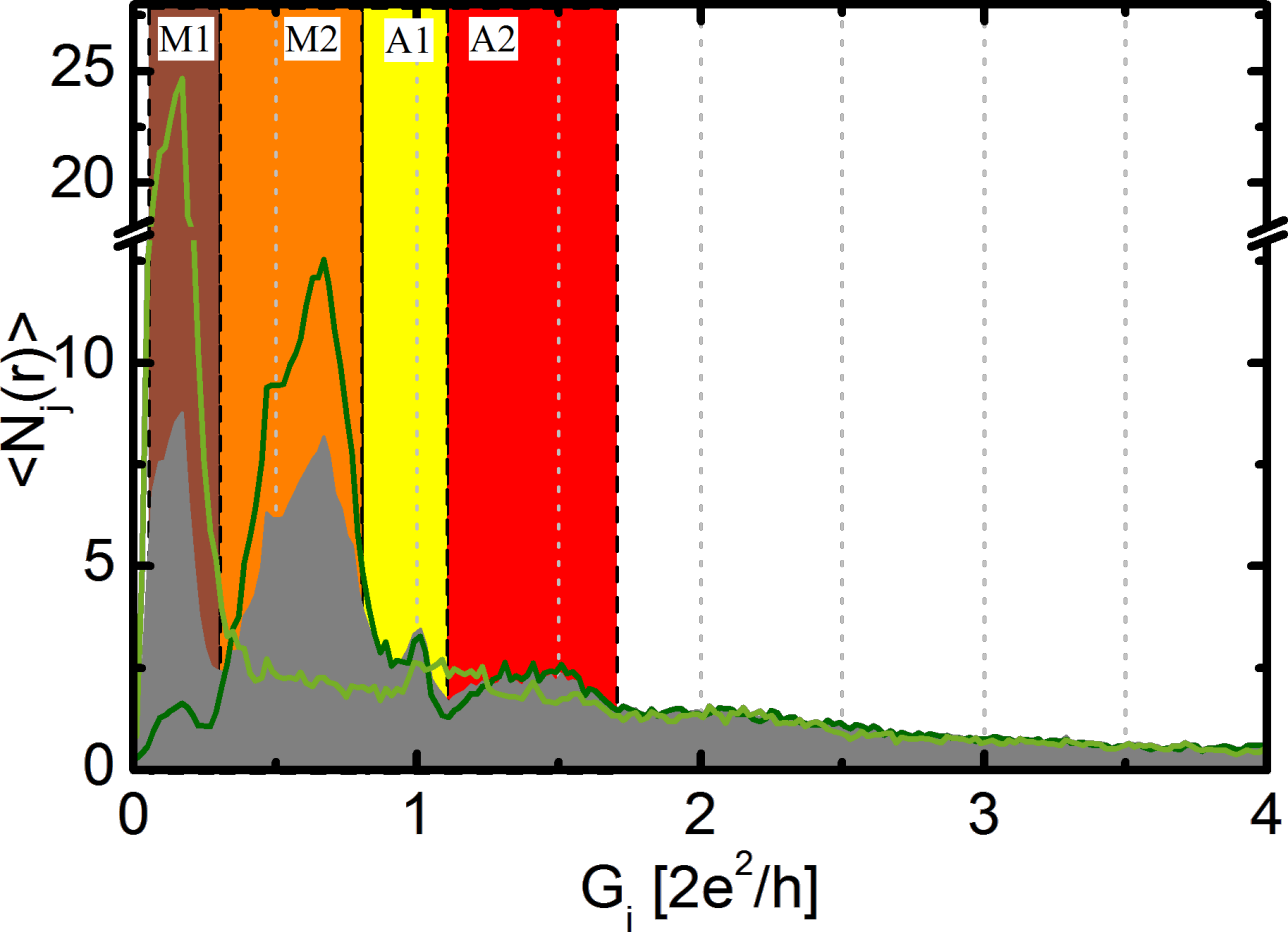
Conditional histograms based on double selection. First the traces are selected for which the molecular plateau in the entire M1–M2 region is longer than average, i.e., the traces corresponding to pronounced molecular junctions. The conditional histogram for these traces (grey area graph) is the same as the green line in Figure 2a. As a next step we select traces from this reduced dataset, for which the M1 (M2) region is longer than average, respectively. The light and dark green curves show the corresponding conditional histograms for the M1 and M2 region, respectively.

To investigate the relation of the two molecular configurations to each other we have selected the opening traces, where the molecular configurations have been formed (green curve in Figure 2a). This accounts to around 40% of all the breaking traces. Afterwards we have constructed the conditional conductance histogram for this reduced dataset for regions M1 and M2 separately, which is shown in Figure 3. In the conditional histogram selected for the M1 region (green) no peak appears in the M2 region, and similarly the conditional histogram for the M2 configuration (dark green) shows an almost complete suppression of the peak in the M1 region. These observations point to a strong anti-correlation between the two molecular configurations: If one molecular configuration is pronounced, the other is practically absent. This is in agreement with the anticorrelation of regions M1 and M2 shown by the blue spots in the 2DCH.

### Conditional two-dimensional conductance–displacement histograms

So far, we have investigated the appearance of different configurations and their relation, but the shape of the individual traces has been discarded in this analysis. Two typical opening traces are shown in Figure 2c, which are quite different in nature. Instead of the investigation of the individual traces we investigate 2D conductance–displacement histograms(2DCDH) [19,21–23]. This method provides information about the conductance and the corresponding breaking length at the same time.

To construct the 2DCDH a reference conductance, *G*^ref^ is chosen and the traces are shifted along the displacement axis, such, that all the curves have this reference conductance at the zero point of the displacement axis. Afterwards, by dividing the displacement and conductance axis into discrete bins, a 2D histogram is constructed, where the number of counts is shown by colors.

We have chosen 1.7*G*_0_ as the reference value, which is the upper boundary of the precursor region, A2. The electrode separation was calibrated by forming atomic chains in clean gold nanojunctions and by measuring the distance of the peaks in the plateau-length histogram [18–19,36]. Further on, we scale the displacement in the units of the Au–Au distance in pure monoatomic chains, which is around 2.6 Å [36]. We have verified this calibration by measuring the tunneling current as a function of the electrode displacement.

**Figure 4:**
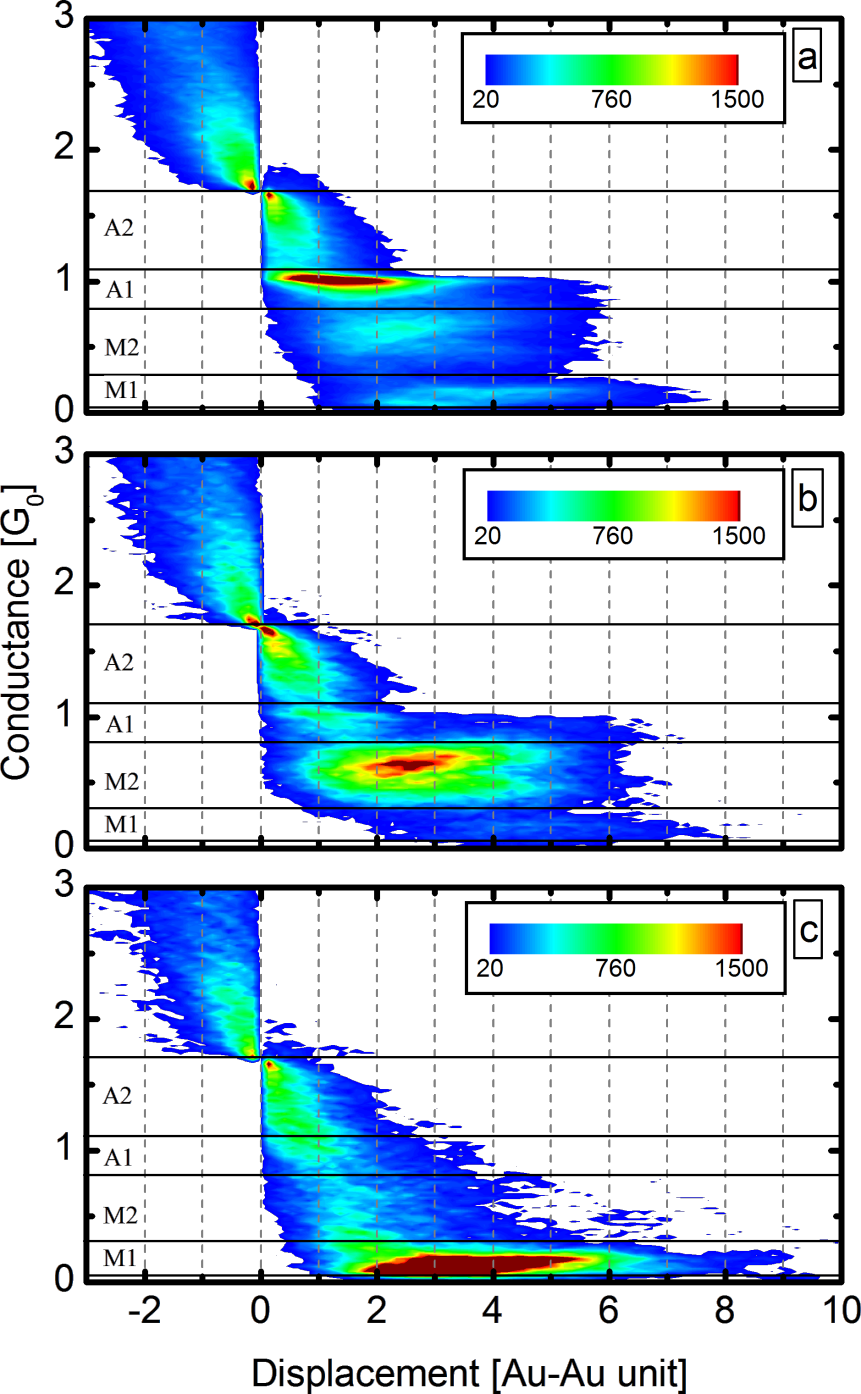
(a) Two-dimensional conductance–displacement histogram for all of the measured traces, showing the appearance of the same configurations as the grey curve in Figure 1. (b,c) The 2D conductance–displacement histograms for the same traces that were used to plot the light/dark green, double selected conditional histograms in Figure 3. Panels (b) and (c) correspond to molecular traces selected for the M2 and M1 region, respectively. The electrode-displacement is given in Au–Au distance unit, i.e., the distance of neighbor Au atoms in clean atomic chains. The 2DCDHs are normalized to the number of included traces.

In Figure 4a the 2DCDH is shown for all traces, demonstrating that the A1, M1 and M2 configurations all have extended plateaus indicating atomic chain formation in all regions. As all the traces are included in this plot, different types of breaking traces can not be distinguished in the 2DCDH. To resolve the typical trajectories, we have investigated the 2DCDH for separate sets of curves.

Figure 4b,c shows the 2DCDHs for the same double-selected datasets that were used to plot the light and dark green conditional histograms in Figure 3, i.e., for molecule-affected opening traces longer than average in the M1 or M2 region. Both plots exhibit an extended plateau in the region of the selection with up to 5–6 Au–Au unit lengths, and a substantial suppression of the other two regions. This points to two distinct types of molecule-assisted chain-formation processes. That is, either a chain with M1 or M2 conductance is formed, but there is no significant transition between these two. We emphasize that this is in clear contrast to our previous observations on Pt–CO junctions [19], where Pt atomic chains can be pulled either by a perpendicular or a parallel CO molecule sitting in the chain, and the molecule frequently rotates from the perpendicular to the parallel orientation along the chain formation process, i.e., a clear transition is observed between the corresponding two conductance intervals.

### Final configuration analysis

**Figure 5:**
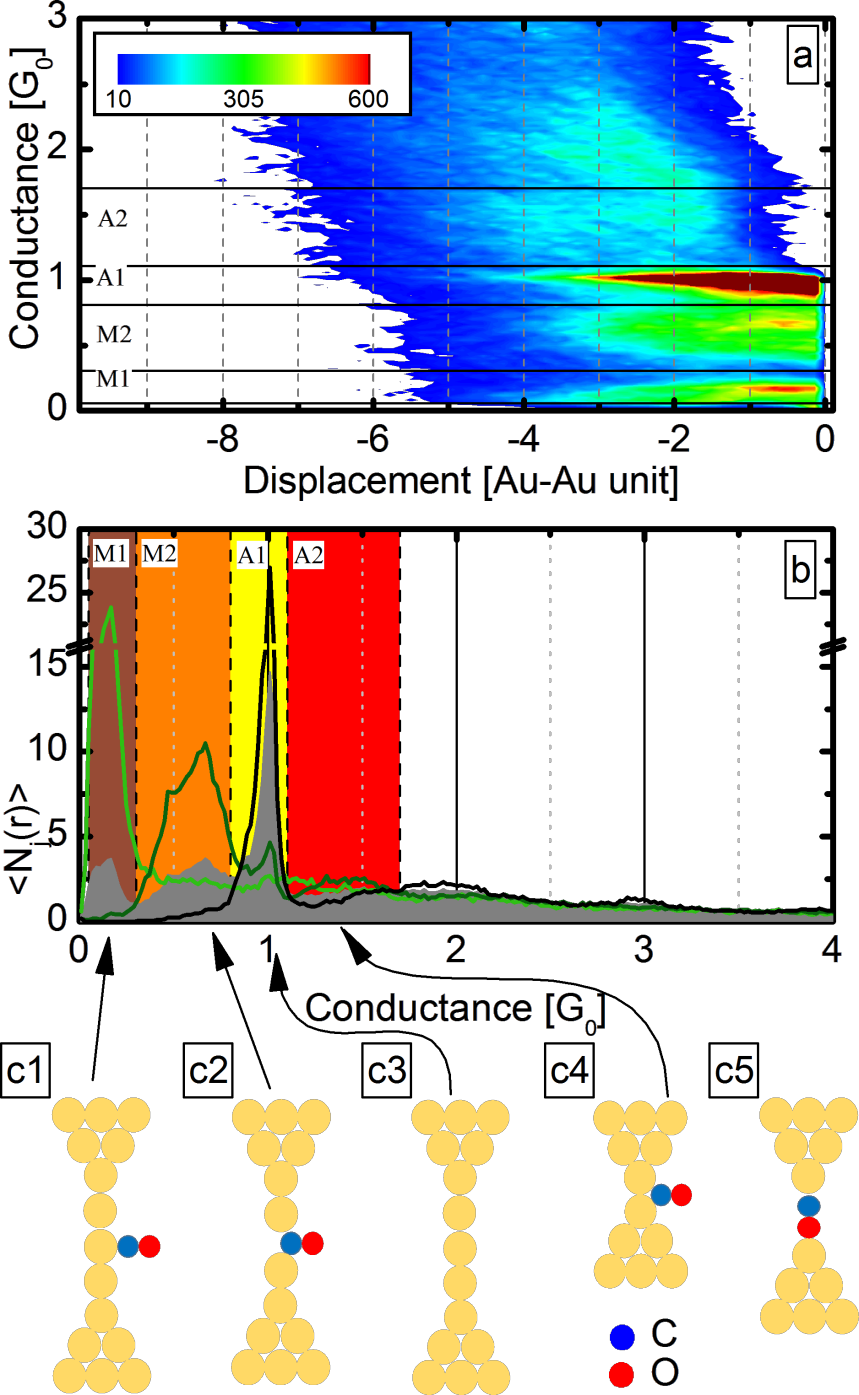
(a) The 2DCDH of all traces with *G*^ref^ = 0.01 *G*_0_. This histogram shows that the junction can break from all the three regions (M1–M2, A1). (b) The histograms of selected traces according to the final configuration selection for the M1 region (light green), M2 region (dark green) and the A1 region (black). (c1–c5) Configurations that can be formed during the junction opening: (c1) and (c2) Au atomic chain with a CO molecule bound in an atop and bridge geometry, respectively. (c3) Pure Au atomic chain. (c4) Precursor molecular configuration. (c5) A parallel CO molecule is incorporated in the chain. (According to our arguments the latter is not observed in the present experiments.)

We note here, that particular features are enhanced in the above 2DCDH analysis. For example, since we have selected long traces for the defining regions all the displayed curves in this region are longer than average. Also the choice of the reference conductance affects the shape of the 2DCDH map. Therefore, the 2DCDH of all traces with different *G*^ref^ is shown in Figure 5a. Here all the curves are fitted together at the breaking point, which we define as *G*^ref^ = 0.01*G*_0_. We can observe that after extended plateaus in the A1, M2 or M1 region the junction can break from all these configurations.

Similarly to our study on Pt–CO junctions[19] it is useful to select the traces according to their final configuration. A trace is considered to break from one of the three contact configurations (A1, M2 or M1) if 70% of the final 30 data points (approx. 1 Å) before the rupture is within the corresponding conductance region. According to this selection, respectively, 42%, 27% and 14% of all traces break from the A1, M2 or M1 region, and for the remaining 17% of the traces our algorithm does not identify a clear final configuration.

The conditional histogram for the selected traces according to their final configuration is shown in Figure 5b. As a reference the grey area graph shows the histogram of all the recorded traces. The final configuration histogram for the M2 region (dark green curve) shows a strong suppression of the M1 and A1 region, and an enhancement of the A2 region. The final configuration histogram of the M1 region (light green) shows a strong suppression of the M2 and A1 region, and it also shows an enhancement of the A2 region, which supports our previous finding: In each single trace only one of the two molecular configurations is pronounced. This analysis also shows, that the M2 molecular configuration has a precursor configuration centered at the middle of the A2 region, whereas the precursor configuration of the M1 configuration has somewhat lower conductance, centered rather at the border of the A1 and A2 regions. The final configuration histogram for the M2 region also shows a minor peak around 1*G*_0_, demonstrating that the M2 configuration is not always preceded by the A2 precursor molecular configuration, but occasionally it is preceded by a pure Au monoatomic contact. The latter observation is also supported by the 2DCDH in Figure 4b.

## Discussion

Our analysis showed that the junctions can break from three different configurations (A1, M1, M2) and on one trace only one out of these three configurations is pronounced with a length up to 5–6 Au–Au unit. This means that three distinct chain formation processes are observed: (i) pure Au monoatomic chains, (ii) molecule affected chains with M2 conductance, (iii) molecule affected chains with M1 conductance. According to our analysis there is no significant transition between these three types of chains.

To identify the M1 and M2 molecular configurations we compare our results to the ab initio simulations in [29–30]. The conductance of the second molecular peak (M2) coincides with the calculated conductance of a bridge-like molecular configuration (0.6–0.7*G*_0_), where the CO molecule sits between two neighboring Au chain atoms in a perpendicular direction with respect to the contact axis (see Figure 5c2), similarly to our observations on Pt–CO junctions [19].

The M1 molecular configuration could be related to another configuration, where the CO molecule sits between two Au atoms in a parallel direction (see Figure 5c5). However, it is reasonable to assume that before this configuration a perpendicular configuration is formed, which is then rotated to the parallel orientation, as it was observed in Pt–CO molecule-decorated chains [19]. In the present experiments no transition between the two molecular configurations is observed, therefore we do not favor the interpretation of the M1 configuration as a parallel CO molecular junction.

As an alternative we consider the so-called atop geometry reported in [29–30], where the CO molecule is not wedged in between two Au atoms, but it binds to the side of a single Au atom of a gold atomic chain (see Figure 5c1). This geometry is interesting, since one would expect perfect transmission for the undistorted Au atomic chain, but the CO molecule binding to the side induces a strong reduction of the conductance due to a Fano-like destructive interference effect. The calculated conductance of this geometry (0.08*G*_0_) [29–30] agrees with the conductance of the M1 region. We favor this interpretation, as it can also account for the absence of transition between the molecule affected chains with M1 and M2 conductance.

Similarly to our previous study on Ag–CO junctions the A2 precursor configuration is considered as a CO molecule bound to the side of a dimer Au junction (see Figure 5c4).

We also note that the M2 configuration exhibits a clear positive slope of the conductance plateau (see Figure 4b and also the second sample trace in Figure 2c), which agrees with [29,37].

## Conclusion

In this paper we have investigated the formation and evolution of Au–CO single-molecule junctions. To get more insight into the junction formation we used different statistical analysis methods, such as correlational analysis, 2D conductance–displacement histograms, and conditional histograms. The combination of these techniques can reveal information far beyond simple conductance histogram measurements. We have observed the formation of two molecular configurations, which can stretch over several atom-atom distances, forming molecule-decorated atomic chains. The evolution of Au–CO atomic chains is in clear contrast to our previous report on Pt–CO molecule-affected chain formation. In the latter case the molecular configuration with higher conductance (perpendicular CO) is transformed to the molecular configuration with lower conductance (parallel CO) along the chain formation. In the present experiments, however, no transition is observed between the two distinct types of molecule-decorated chains. This behaviour is naturally explained if the low conductance molecular configuration is not a derivative of the high conductance one, rather it is a completely different structural arrangement. The two simulated molecular configurations in [29–30] might account for our observations, as the higher conductance of the bridge geometry and Fano-interference-suppressed lower conductance of the structurally different atop geometry coincide with the experimentally observed peak positions. A precursor molecular configuration was also observed, from which the molecular junctions are likely to be formed.

## Experimental

The measurements were performed with a custom-built MCBJ setup at liquid helium temperature and the CO molecules were dosed with a custom-built vacuum system from a high purity container through a heated tube. The detailed description of our experimental technique is introduced in our previous publication [27].

We have performed our measurements on one break-junctions sample that was measured for several weeks as follows. In order to exclude unwanted contamination we have a very strict protocol for this type of measurement. First we bake out and pump the sample holder for approximately one week. Afterwards we cool down the sample holder and record clean histograms. As a next step we precisely mimic all steps of molecule dosing (heating of the dosing tube, opening the shutter and the proper valves, etc.) except that the gas container remains closed and we check that the clean metal histogram is preserved. After the real dosing of the molecules, new peaks appeared and were present at moderate bias voltage (approx. 30–50 mV) for a long time (10,000–20,000 opening–closing cycles) without further dosing. Then we could permanently desorb the molecules through a high bias voltage (≥100 mV) histogram measurement. With this step, a clean Au histogram was reestablished, and no molecular peaks were observed even if the bias voltage was reduced back (approx. 30–50 mV), so a new dosing was necessary to see molecular peaks again. In the present study three independent data sets were acquired by permanent desorption of the CO molecules and repeated dosing, which show reproducible data.

The displacement of the electrodes was calibrated prior to molecule dosing using both plateau-length histograms and conductance traces in the tunneling region. To ensure proper cleanliness during the molecular measurements the turbomolecular pump was continuously running. This has introduced vibrations on a scale below 1 Å to the junction, which resulted in the appearance of false peaks in the plateaus-length histograms of molecular junctions, thus we have avoided drawing conclusions from the plateau-length histograms of the molecules.
